# Impact of Age at Narcolepsy Onset on Sleep-Onset REM Periods in the Multiple Sleep Latency Test

**DOI:** 10.3390/jcm14124379

**Published:** 2025-06-19

**Authors:** Jun-Sang Sunwoo, Ki-Hwan Ji, Daeyoung Kim, Kyung Min Kim, Yun Ho Choi, Jae Wook Cho, Hyeyun Kim, Wonwoo Lee, Yu Jin Jung, Dae Lim Koo, Hee-Jin Im, Kwang Ik Yang

**Affiliations:** 1 Department of Neurology, Kangbuk Samsung Hospital, Seoul 03181, Republic of Korea; ultrajs4@gmail.com; 2 Department of Neurology, Inje University Busan Paik Hospital, College of Medicine, Inje University, Busan 47392, Republic of Korea; kihwanji@gmail.com; 3 Department of Neurology, Chungnam National University Hospital, Chungnam National University College of Medicine, Daejeon 35015, Republic of Korea; bigbread.kim@gmail.com; 4 Department of Neurology, Severance Hospital, Yonsei University College of Medicine, Seoul 03722, Republic of Korea; penlighting11@yuhs.ac; 5 Department of Neurology, Incheon St. Mary’s Hospital, College of Medicine, The Catholic University of Korea, Seoul 06591, Republic of Korea; recluse21@naver.com; 6 Department of Neurology, Pusan National University Yangsan Hospital, Pusan National University College of Medicine, Yangsan 50612, Republic of Korea; medofficer@hanmail.net; 7 Department of Neurology, International St. Mary’s Hospital, Catholic Kwandong University College of Medicine, Incheon 22711, Republic of Korea; imkhy77@gmail.com; 8 Department of Neurology, Yongin Severance Hospital, Yonsei University College of Medicine, Yongin 16995, Republic of Korea; nawonwoo01@yuhs.ac; 9 Department of Neurology, Kyung Hee University Hospital at Gandong, College of Medicine, Kyung Hee University, Seoul 05278, Republic of Korea; eugene0517@gmail.com; 10 Department of Neurology, Seoul Metropolitan Government Seoul National University Boramae Medical Center, Seoul National University College of Medicine, Seoul 07061, Republic of Korea; koodaelim@gmail.com; 11 Department of Neurology, Dongtan Sacred Heart Hospital, Hallym University Medical Center, College of Medicine, Hwaseong 18450, Republic of Korea; coolere@naver.com; 12 Center for Sleep and Brain Health, Department of Neurology, Soonchunhyang University College of Medicine, Cheonan Hospital, Cheonan 31151, Republic of Korea

**Keywords:** narcolepsy, age of onset, REM sleep latency, multiple sleep latency test

## Abstract

**Background/Objectives:** This study aimed to investigate the effect of age at symptom onset on rapid eye movement (REM) sleep latency and sleep-onset REM period (SOREMP) distribution in multiple sleep latency tests (MSLTs) in patients with narcolepsy. **Methods**: This was a retrospective multicenter chart review of 135 newly diagnosed drug-naïve patients with narcolepsy who underwent MSLT and fulfilled the diagnostic criteria for narcolepsy. The age at onset was defined as the first occurrence of excessive daytime sleepiness or cataplexy. We investigated sleep onset latency, REM sleep latency, and the presence of SORMEP in each nap trial of the MSLT. The clinical, polysomnography, and MSLT findings were compared between the early- and late-onset groups. Correlation and linear regression analyses were used to assess the effect of age at onset as a continuous variable, and survival analyses confirmed its impact on the MSLT parameters. **Results**: The mean age at onset was 18.3 ± 8.8 years. Patients with early onset had a higher rate of SOREMPs than late-onset patients in the first MSLT nap (81.9% vs. 63.3%, *p* = 0.031). However, the severity of the narcolepsy symptoms did not differ between the groups. In linear regression analysis, age at onset was significantly associated with MSLT REM sleep latency (β = 0.049, *p* = 0.033) after adjusting for confounders. Survival analysis confirmed that an early onset of narcolepsy was associated with a higher probability of SOREMPs in the first MSLT nap (hazard ratio 0.955, *p* = 0.001). **Conclusions**: A younger age at narcolepsy onset was associated with shorter REM sleep latency and higher SOREMP probability in MSLT. These findings indicate that the early onset of narcolepsy may be linked to greater disease severity in terms of REM sleep dysregulation.

## 1. Introduction

Narcolepsy is a chronic neurological disorder characterized by excessive daytime sleepiness (EDS). Other clinical symptoms include cataplexy, hypnagogic and hypnopompic hallucinations, sleep paralysis, and disrupted nocturnal sleep [1]. In the third edition of the International Classification of Sleep Disorders, narcolepsy is classified into two subtypes: Type 1 (NT1) and Type 2 (NT2). NT1 is characterized by low hypocretin levels in the cerebrospinal fluid (CSF), and almost all patients have NT1 exhibit cataplexy. However, patients with NT2 have normal CSF hypocretin levels (i.e., either >110 pg/mL or >1/3 of mean normal values) and lack cataplexy [2].

The global prevalence of narcolepsy is estimated to range from 0.02% to 0.05% [3]. The prevalence of narcolepsy with cataplexy in Korean adolescents was reported to be 0.015% [4]. The onset of narcolepsy symptoms mostly occurs in adolescents and young adults. The first manifestations of daytime sleepiness and cataplexy peaked between 15 and 19 years of age, while those of sleep paralysis and hypnagogic hallucinations peaked between 20 and 24 years of age [5]. A previous study from Canadian and French cohorts demonstrated a bimodal distribution of age at onset of narcolepsy: the first peak at 14.7 years and the second peak at 35 years [6]. Nationwide estimates for narcolepsy in Korea showed similar findings, with the incidence peaking at 15–19 years, followed by 20–24, 25–29, 30–34, and 10–14 years [7]. Age at onset has been proposed as a potential modifier of disease expression, with earlier onset cases potentially reflecting a more genetically driven or severe phenotype [6]. Moreover, previous studies reported that the clinical phenotype of narcolepsy changes with age. As patients with narcolepsy age, both the degree of daytime sleepiness and the frequency of cataplexy decreased, and the MSLT abnormalities also became milder [8,9].

However, little is known about how age at symptom onset affects objective sleep architecture, particularly sleep-onset REM periods (SOREMPs) during the multiple sleep latency test (MSLT). The number and timing of SOREMPs in the MSLT are critical electrophysiological markers of REM sleep dysregulation in narcolepsy [3]. If the age at onset is involved in the pathogenesis of narcolepsy, it is plausible that it may influence the occurrence of SOREMPs during the MSLT. To date, only a limited number of studies have specifically examined this relationship, and existing evidence remains inconclusive. Two previous studies [6,10] consistently reported that younger age at onset was associated with shorter mean sleep latency on the MSLT but not with the number of SOREMPs. However, they did not examine the distribution or timing of SOREMPs across individual nap trials, which might provide additional insight into the effect of age at onset on SOREMP in the MSLT.

The present study aimed to address this gap by investigating the association between age at narcolepsy onset and REM sleep latency and SOREMP distribution in the MSLT, as well as the clinical characteristics and PSG findings.

## 2. Materials and Methods

We constructed a multicenter cohort of 137 patients newly diagnosed with narcolepsy across 11 medical institutions in South Korea between 2011 and 2024. We then performed a retrospective chart review to extract clinical information and diagnostic test results from the patients’ medical records. All patients enrolled in this cohort presented with EDS with or without cataplexy, underwent overnight PSG and MSLT for diagnostic evaluation, and fulfilled the diagnostic criteria for NT1 or NT2 according to the International Classification of Sleep Disorders (ICSD), third edition. Because CSF hypocretin measurements are not available in South Korea, none of the patients underwent CSF studies, and therefore, all had to be confirmed as positive for the MSLT (both mean sleep latency ≤ 8 min and ≥2 sleep-onset rapid eye movement periods [SOREMPs] on the MSLT and overnight PSG). Subsequently, patients were classified as having NT1 if they exhibited cataplexy and NT2 if cataplexy was absent.

In preparation, the sleep–wake schedule was checked using a sleep diary with or without actigraphy for two weeks prior to the MSLT to exclude insufficient sleep syndrome, delayed sleep–wake phase disorder, or other secondary causes of EDS. A habitual sleep duration of at least 6 h was required to exclude insufficient sleep syndrome as a cause of EDS. In cases accompanied by delayed sleep–wake phase disorder, which is particularly common in adolescents and young adults, the circadian phase was corrected before the MSLT; if not corrected, the MSLT was performed in accordance with the patient’s habitual sleep–wake schedule to avoid confounding by the circadian phase. Other sleep disorders were defined according to the diagnostic criteria of the ICSD, 3rd edition, and the decision to perform PSG and MSLT was made at the discretion of each investigator. All investigators participating in this study were experienced neurologists and sleep medicine specialists with extensive expertise in diagnosing sleep disorders, including narcolepsy. Overnight PSG followed by MSLT was performed according to the protocol recommended by the American Academy of Sleep Medicine (AASM) to ensure accuracy and reliability [11]. The study protocol was approved by the Institutional Review Board of Soonchunhyang University Cheonan Hospital (SCHCA 2023-08-042) and conducted in accordance with the Declaration of Helsinki and Good Clinical Practice guidelines. The requirement for written informed consent was waived because only the medical records were retrospectively collected.

Sleep stages and associated events were scored in 30 s epochs according to the AASM manual version 2.1. We investigated PSG findings, including total sleep time, sleep latency, rapid eye movement (REM) sleep latency, sleep efficiency, wakefulness after sleep onset (WASO), percentage of sleep stages (N1, N2, N3, and REM sleep), total arousal index, the apnea–hypopnea index (AHI), mean and minimum oxygen saturation measured by pulse oximetry (SpO2), and the periodic limb movement index (PLMI). Regarding the MSLT data, we investigated the sleep onset latency, REM sleep latency, and the presence of SORMEP in each nap trial. The mean sleep latency was calculated by averaging the sleep onset latency of all nap trials, with the sleep latency of a nap trial that ended before sleep recorded as 20 min. The REM sleep latency was defined as the interval between the first epoch of any sleep stage and the first epoch of REM sleep. Additionally, the MSLT REM sleep latency was averaged only across the nap trials in which REM sleep was present. SOREMP was defined as positive if REM sleep occurred within 15 min of sleep onset in each nap trial. Five-nap trials were performed in 123 (91.1%) patients, and the remaining 12 (8.9%) underwent four-nap trials for MSLT. The 4-nap MSLT was performed only when the results of the four naps clearly met the diagnostic criteria for narcolepsy.

A retrospective chart review was performed to extract clinical information at the time of diagnosis (i.e., positive MSLT) and before starting pharmacological treatment for narcolepsy. The age at onset of narcolepsy was defined as the age at which either EDS or cataplexy first occurred. The age at diagnosis was defined as the age at which a positive MSLT result for narcolepsy was confirmed. We also evaluated sex, narcolepsy type, the body mass index (BMI), smoking, coffee and caffeine intake, the Epworth Sleepiness Scale (ESS) score, the Pittsburgh Sleep Quality Index (PSQI) score, the presence of cataplexy, hypnagogic/hypnopompic hallucinations, and sleep paralysis. The symptom tetrad was defined as the presence of all four cardinal symptoms of narcolepsy (EDS, cataplexy, hallucinations, and sleep paralysis), whereas the triad was defined as the presence of three cardinal symptoms. Two patients without information on age at onset were excluded, and 135 patients with narcolepsy were included in this study.

The normality of each continuous variable was assessed using the Shapiro–Wilk test. For BMI and REM percentage, which followed a normal distribution, we used Student’s *t*-test to compare the means between the two groups. The remaining variables violated the assumption of normality, and the nonparametric Mann–Whitney U test was used instead. The distributions of categorical variables were compared using the chi-square or Fisher’s exact test, as appropriate. To identify variables associated with age at onset, we first conducted a correlation analysis using continuous variables from PSG, MSLT, and clinical data. As the age at onset did not meet the assumption of normality, Spearman’s correlation coefficient (ρ) was used to measure the linear correlation with other continuous variables. Based on this result, we subsequently performed univariate and multivariate linear regression analyses, with MSLT REM sleep latency being the dependent variable, and estimated the regression coefficient (β) and 95% confidence interval (CI). Variables showing significant associations in the univariate analysis were included in the multivariate model, along with categorical variables with clinical relevance, such as sex and narcolepsy type.

Additionally, we performed survival analysis to examine the relationship between age at onset and REM sleep latency in the MSLT. Time-to-event or survival time was defined as REM sleep latency (i.e., the time from sleep onset to REM sleep onset) in each nap trial. As the occurrence of SOREMPs was observed for 15 min after sleep onset, nap trials in which REM sleep did not occur were censored at 15 min (i.e., the end of the nap trial). Nap trials that ended without sleep onset were excluded from the survival analyses. The Kaplan–Meier method was used to estimate the survival function (i.e., the probability of SOREMPs during nap trials), and the log-rank test was used to compare the survival curves between the two age groups. The Cox proportional hazards model was used to estimate hazard ratios (HRs) and 95% CI for their association with the probability of SOREMPs on MSLT. Statistical significance was defined as a two-tailed *p*-value < 0.05. All statistical analyses were performed using SPSS software (version 26, IBM Corp., Armonk, NY, USA).

## 3. Results

**Figure 1:**
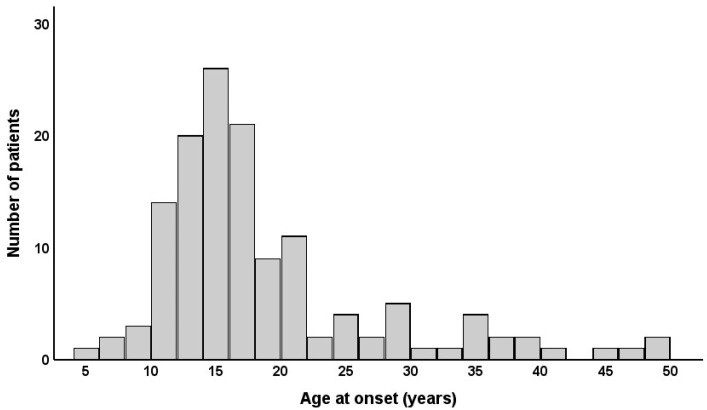
Distribution of age at onset in patients with narcolepsy.

The mean age at diagnosis was 28.1 ± 13.2 years, and 79 (58.5%) patients were male. The mean ESS score at diagnosis was 15.8 ± 3.9. Seventy (51.9%) patients were diagnosed with NT1 and sixty-five (48.1%) were diagnosed with NT2. The mean and median ages at onset were 18.3 ± 8.8 and 16 (interquartile range, 13–20) years old, respectively. The age at onset showed a right-tailed distribution, with a single peak at 15 years (Figure 1). Although the median age at onset was 16 years old, the majority were clustered between the ages of 10 and 20 years old (interquartile range, 13–20 years old). Therefore, we divided the early- and late-onset groups using 20 years old as the cutoff point (i.e., early onset ≤ 20 years old and late onset > 20 years old). According to the narcolepsy type, the mean age at onset of patients with NT1 was 17.0 ± 8.5 years old, which was younger than that of patients with NT2 (19.7 ± 8.9 years old, *p* = 0.013).

Narcolepsy types, ESS scores, and the prevalence of cataplexy, hypnagogic/hypnopompic hallucinations, and sleep paralysis did not differ between patients with early- and late-onset narcolepsy (Table 1). The narcolepsy symptom tetrad and triad were present in 17.8% and 23.0% of the patients, respectively, and did not differ between the two age groups. There were no differences in sex, the BMI, smoking, coffee and caffeine intake, or PSQI scores between the two age groups.

When comparing the MSLT results, the mean sleep latency, number of SOREMP, and REM sleep latency did not differ between the two age groups (Table 2). When comparing each nap trial, the early-onset group had a higher rate of SOREMPs than the late-onset group during the first nap (81.9% vs. 63.3%, *p* = 0.031). However, in the remaining four naps, there was no difference in the SOREMP rate between the two groups. On overnight PSG data, patients with early onset had shorter sleep latency, higher arousal index and AHI, and lower minimum SpO2 than those with late onset (Table 2). However, other PSG parameters, including TST, REM sleep latency, the presence of SOREMP, REM percentage, sleep efficiency, and PLMI, did not differ between the two age groups.

**Figure 2:**
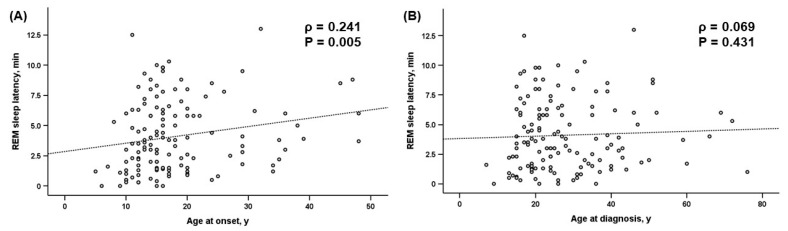
A correlation analysis of REM sleep latency on the MSLT. (**A**) Age at onset. (**B**) Age at diagnosis. ρ denotes Spearman’s correlation coefficient. Abbreviations: REM, rapid eye movement; MSLT, multiple sleep latency test.

In a correlation analysis with age at onset as a continuous variable, we found a significant positive correlation between age at the onset of narcolepsy and REM sleep latency in the MSLT (ρ = 0.241, *p* = 0.005; Figure 2A). However, the mean sleep latency and number of SOREMPs on the MSLT and ESS scores did not correlate with the age at onset. There was no significant correlation between age at onset and PSG parameters, except for N3 percentage (ρ = −0.359, *p* < 0.001) and total arousal index (ρ = 0.200, *p* = 0.020; Table 3). Notably, unlike the age at onset, the age at diagnosis of narcolepsy did not correlate with REM sleep latency in the MSLT (ρ = 0.069, *p* = 0.431; Figure 2B).

Next, we performed a linear regression analysis using MSLT REM sleep latency as the dependent variable. In the univariate analysis, in addition to the age at onset of narcolepsy, the BMI, PSG parameters including sleep efficiency, WASO, N1 percentage, and MSLT parameters including mean sleep latency and the number of SOREMPs were significantly associated with REM sleep latency in the MSLT. Because patients with NT1 had shorter MSLT REM sleep latency than those with NT2 (2.8 ± 2.4 min vs. 5.5 ± 2.9 min, *p* < 0.001), the narcolepsy type was included as a covariate. Moreover, as sleep efficiency, WASO, and N1 percentage are PSG parameters that reflect sleep quality and are related to each other, sleep efficiency was only included as a covariate in the multivariate analysis. In the multiple linear regression analysis, age at onset was significantly associated with MSLT REM sleep latency after adjusting for covariates (β = 0.049, 95% CI 0.004–0.095, *p* = 0.033; Table 4). In addition, the BMI (β = −0.106, 95% CI −0.194–−0.018), NT1 (β = −1.083, 95% CI 1.968–−0.199), the MSLT mean sleep latency (β = 0.258, 95% CI 0.044–0.472), and the number of SOREMPs (β = −0.921, 95% CI −1.335–−0.507) were associated with MSLT REM sleep latency.

**Figure 3:**
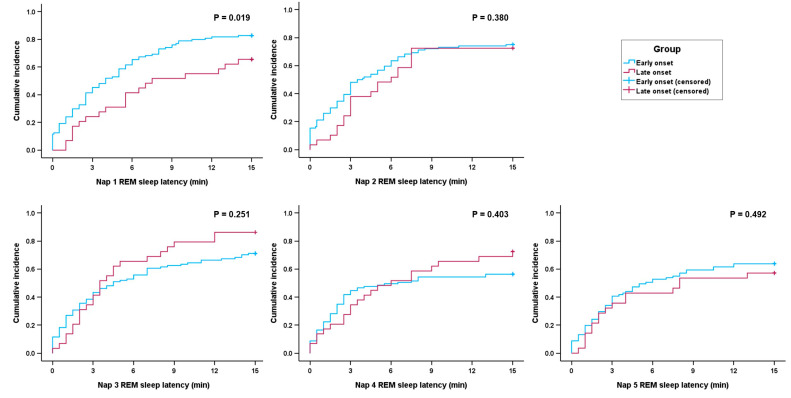
Kaplan–Meier survival plots showing the cumulative incidence of sleep-onset REM periods for each nap trial of the MSLT. Early onset was defined as an age of onset ≤ 20 years, and late onset was defined as an age of onset > 20 years. *p* values were calculated using the log-rank test. Zero on the x-axis indicates sleep onset. Abbreviations: REM, rapid eye movement; MSLT, multiple sleep latency test.

We performed survival analysis to confirm the association between age at onset and MSLT REM sleep latency. Kaplan–Meier survival curves showed that the early-onset group was more likely to have SOREMPs than the late-onset group in the first nap trial (*p* = 0.019 by log-rank test, Figure 3). However, there was no significant association between the age at onset and the probability of SOREMPs in other nap trials.

In the univariate Cox regression analysis, the SOREMP probability in the first nap trial was significantly associated with age at onset (HR 0.966, 95% CI 0.941–0.991, *p* = 0.009), NT1 (HR 1.718, 95% CI 1.166–2.532, *p* = 0.006), the MSLT mean sleep latency (HR 0.858, 95% CI 0.771–0.954, *p* = 0.005), and the number of SOREMPs (HR 2.121, 95% CI 1.719–2.617, *p* < 0.001; Table 5). The multivariate Cox proportional hazards model adjusted for covariates demonstrated that age at onset (HR 0.955, 95% CI 0.929–0.982, *p* = 0.001) and the number of SOREMPs on the MSLT (HR 2.155, 1.707–2.720, *p* < 0.001) were associated with a higher probability of SOREMPs in the first nap trial. In subgroup analysis by narcolepsy type, these associations remained significant in both the NT1 and NT2 subgroups (Table S1).

## 4. Discussion

In the present study, the age at onset of narcolepsy was significantly correlated with REM sleep latency on the MSLT, indicating that the younger the age of onset, the earlier SOREMPs occurred during MSLT naps. The correlation remained significant even after adjusting for confounding factors, such as the number of SOREMPs, narcolepsy type, and PSG sleep efficiency, supporting the independent association between age of onset and MSLT REM sleep latency. A shorter MSLT REM sleep latency suggests more severe impairment in REM sleep regulation in patients with narcolepsy. Hypocretin (or orexin) neurons suppress REM sleep during wakefulness by inhibiting REM-on neurons and activating REM-off neurons [12]. Therefore, hypocretin deficiency in narcolepsy leads to the disinhibition of REM sleep and enhances propensity to enter REM sleep [13]. In this regard, patients with early-onset narcolepsy may have more severe REM sleep dysregulation than those with late-onset narcolepsy. This is consistent with a previous study showing that an early age of onset was associated with a higher frequency of cataplexy and a positive family history than a late age of onset [6]. It was speculated that the age at onset of narcolepsy is genetically determined, and a younger age at onset is associated with greater disease severity. Similarly, in other neurological and psychiatric diseases, such as Alzheimer’s disease, major depressive disorder, and bipolar disorder, early age at onset is associated with more severe clinical features, a more aggressive disease course, and larger genetic predisposition than a late age at onset [14,15,16]. However, the relationship between the age at onset of narcolepsy and the severity of CSF hypocretin deficiency has not been determined. In addition, our finding that the age of onset did not affect any clinical symptoms of narcolepsy, including cataplexy, hallucinations, or sleep paralysis, contradicts this hypothesis. Nevsimalova et al. also found no association between age at onset and the clinical severity of narcolepsy [10]. Further studies are needed to confirm the effect of age at narcolepsy onset.

A previous study on MSLT in patients with narcolepsy showed that the number of SOREMPs decreased and mean sleep latency increased with age [9]. The authors hypothesized that the neural mechanisms responsible for SOREMPs may weaken with age in narcolepsy patients. However, they also suggested a possibility that these MSLT changes resulted from normal aging. A higher number of SOMREMPs in adolescents than in older patients with narcolepsy and suspected hypersomnia has also been observed in other studies [17,18]. These results suggest that more SOREMPs in early-onset narcolepsy may reflect age-related changes in MSLT. The mean ages at symptom onset and diagnosis of narcolepsy were 18.3 and 28.1 years, respectively, in our study. The diagnostic delay of approximately 10 years was consistent with those reported in previous studies [19,20]. As age at onset is inevitably and closely related to age at diagnosis, the results of our study cannot completely rule out the influence of aging itself. However, it should be noted that the REM sleep latency on the MSLT was not correlated with age at diagnosis but with age at onset, supporting the notion that this correlation is not simply attributed to an effect of aging.

Narcolepsy type is a potential confounder in the association between age at onset and SOREMPs in MSLT. NT1 has a distinct clinical phenotype and pathophysiology compared with NT2. In one narcolepsy cohort, NT1 patients had a significantly higher rate of ≥3 SOREMPs in MSLT than NT2 patients (59.2% vs. 26.9%) [21]. NT1 was associated with more SOREMPs and shorter REM sleep latency on the MSLT than NT2 [22,23], which was also confirmed in the present study. However, in the multivariate analysis, age at narcolepsy onset remained significantly associated with MSLT REM sleep latency, even after adjusting for specific narcolepsy types (i.e., NT1 vs. NT2). Furthermore, survival analyses for positive SOREMP in the first nap of the MSLT showed significant associations with age at onset in both the NT1 and NT2 subgroups. These findings suggest that age at onset may have an independent association with REM sleep latency or SOREMPs in the MSLT, beyond the influence of narcolepsy type.

Moreover, owing to the lack of solid biomarkers, there is a potential risk of false positive results in the diagnosis of NT2. To address this concern, we conducted a subgroup analysis by separating the NT1 and NT2 subgroups, which unfortunately failed to reach significant differences between the two age groups. Subgroup analyses inevitably reduced the sample size, making it difficult to achieve a sufficient statistical power. Therefore, future studies with larger datasets focusing on NT1 patients are necessary to confirm these results.

Sansa et al. reported that SOREMPs in the MSLT were not evenly distributed in patients with narcolepsy: the probability of SOREMPs was the highest in the first nap (74.4%) and lowest in the fourth nap (53.4%) [24]. These results are consistent with the distribution of SORMEPs in our study: 77.8% in the first nap and 58.5% in the fourth nap. The authors attributed this phenomenon to a decrease in REM sleep propensity in the afternoon. They also found that younger narcoleptics had shorter REM sleep latency and more SOREMPs in the MSLT than older narcoleptics; however, the effect of age on each nap trial was not determined.

Another notable finding of our study was that an increase in the incidence of SOREMPs in patients with early-onset narcolepsy was observed only in the first nap of the MSLT. Similarly, the survival curves for SOREMP probability between the early- and late-onset groups were significantly different only in the first nap trial. The REM sleep propensity has physiological diurnal variation: it is highest in the early morning and gradually decreases until the evening [25]. Therefore, if the age of onset affects REM sleep dysregulation in narcolepsy, it is likely to be most pronounced during the first nap of MSLT, which corresponds to early morning. REM sleep propensity is tightly regulated by circadian rhythms [26]. A delayed sleep–wake cycle, which is common in adolescents, may have contributed to more SOREMPs and shorter REM sleep latency in the first nap trial of the MSLT in young patients with narcolepsy. To prevent potential confounding by a delayed sleep–wake phase, we adjusted the timing of PSG termination and MSLT initiation to match an individual’s habitual sleep–wake schedule.

The strength of the current study is the use of survival analysis to investigate the association between age at the onset of narcolepsy and MSLT REM sleep latency. The presence of SOREMPs on the MSLT is typical time-to-event data. Therefore, when using correlation or regression analyses for REM sleep latency, nap trials in which REM sleep did not occur (i.e., censored data) must be excluded. However, survival analysis allows for the more accurate modeling of MSLT REM sleep latency by handling censored data [27,28].

This study had several limitations. First, the diagnosis of NT1 was based on the presence of cataplexy and positive MSLT findings because CSF hypocretin measurements were not available. The absence of CSF hypocretin testing may have contributed to the high NT2 rate observed in this study. Although we performed PSG and MSLT according to the standard protocol to ensure a reliable diagnosis of narcolepsy, the possibility of false-positive diagnosis among NT2 patients cannot be ruled out. Nocturnal SOREMPs in the overnight PSG and ≥ 4 SOREMPs in the MSLT were found in 26.2% (17/65) and 29.2% (19/65) of NT2 patients, respectively. The frequencies were significantly lower than those in NT1 patients (54.3% and 61.4%, respectively; *p* < 0.001). However, there was no information on other supporting evidence for narcolepsy, such as CSF hypocretin levels, HLA typing, the sleep stage sequence of SOREMPs, or MSLT retest results, which is an inherent limitation of this retrospective chart review study. Second, due to the retrospective chart review design of this study, some clinical information was either unavailable or incomplete. Disturbed nocturnal sleep, another cardinal symptom of narcolepsy, was not investigated and the severity of narcolepsy symptoms was not comprehensively measured, with the exception of EDS. Third, the age of symptom onset relied on self-reporting and was, therefore, inherently susceptible to recall bias. The diagnostic delay in our study was approximately 10 years, which may have increased the risk of bias. Furthermore, although diagnostic delay may have influenced the MSLT findings, it was not included as a variable in our analysis, which is another limitation of this study. Finally, although 135 patients with narcolepsy were enrolled, the number of patients in the late-onset group was relatively low (30 patients). However, it should be noted that the influence of age at onset on the MSLT REM sleep latency was also confirmed as a continuous variable.

## 5. Conclusions

Age at onset was positively correlated with REM sleep latency in the MSLT in patients with narcolepsy. Furthermore, the effect of age at narcolepsy onset on SOREMPs was most pronounced during the first nap of the MSLT. Our results suggest that early onset of narcolepsy may be associated with great disease severity, specifically related to REM sleep dysregulation. However, we cannot rule out the possibility that these results may reflect age-related changes in the MSLT; therefore, caution is required when interpreting the results. Further studies are required to explore the potential underlying mechanisms linking age at onset and REM sleep dysregulation in patients with narcolepsy.

## Figures and Tables

**Table 1:** Demographic and clinical information.

Variables	Total (*n* = 135)	Early Onset (*n* = 105)	Late Onset (*n* = 30)	*p*
Age at onset, y	18.3 ± 8.8	14.4 ± 3.3	32.0 ± 8.1	<0.001
Age at diagnosis, y	28.1 ± 13.2	24.8 ±12.0	39.7 ± 10.2	<0.001
Sex, male	79 (58.5%)	61 (58.1%)	18 (60.0%)	0.852
Narcolepsy type				0.141
NT1	70 (51.9%)	58 (55.2%)	12 (40.0%)	
NT2	65 (48.1%)	47 (44.8%)	18 (60.0%)	
BMI, kg/m^2^	25.1 ± 4.5	25.2 ± 4.8	24.9 ± 3.5	0.930
Current smoker (*n* = 134)	39 (29.1%)	29 (27.6%)	10 (34.5%)	0.471
Coffee (*n* = 126)	78 (61.9%)	58 (58.6%)	20 (74.1%)	0.142
Caffeine drink (*n* = 76)	20 (26.3%)	15 (25.9%)	5 (27.8%)	1
ESS score (*n* = 132)	15.8 ± 3.9	15.7 ± 3.8	16.1 ± 4.5	0.689
Nap (*n* = 129)	113 (87.6%)	88 (88.0%)	25 (86.2%)	0.756
PSQI score (*n* = 104)	10.7 ± 5.3	11.1 ± 5.5	9.3 ± 4.5	0.136
PSQI score > 5	93 (89.4%)	73 (91.3%)	20 (83.3%)	0.273
Cataplexy	70 (51.9%)	58 (55.2%)	12 (40.0%)	0.141
Hallucination (*n* = 114)	46 (40.4%)	39 (43.8%)	7 (28.0%)	0.154
Sleep paralysis (*n* = 113)	52 (46.0%)	41 (47.1%)	11 (42.3%)	0.665
Tetrad	24 (17.8%)	20 (19.0%)	4 (13.3%)	0.470
Triad	31 (23.0%)	26 (24.8%)	5 (16.7%)	0.353

Data are presented as mean ± standard deviation or number (%). Abbreviations: NT1, narcolepsy type 1; NT2, narcolepsy type 2; BMI, body mass index; ESS, Epworth Sleepiness Scale; PSQI, Pittsburgh Sleep Quality Index.

**Table 2:** Polysomnography and multiple sleep latency test data.

Variables	Total (*n* = 135)	Early Onset (*n* = 105)	Late Onset (*n* = 30)	*p*
**MSLT**				
Mean sleep latency, min	2.8 ± 2.1	2.9 ± 2.2	2.8 ± 1.7	0.518
No. of SOREMPs	3.4 ± 1.1	3.4 ± 1.1	3.5 ± 1.1	0.732
Positive SOREMP				
1st nap	105 (77.8%)	86 (81.9%)	19 (63.3%)	0.031
2nd nap	99 (73.3%)	78 (74.3%)	21 (70.0%)	0.640
3rd nap	99 (73.3%)	74 (70.5%)	25 (83.3%)	0.160
4th nap	79 (58.5%)	58 (55.2%)	21 (70.0%)	0.148
5th nap (*n* = 123)	74 (60.2%)	58 (61.7%)	16 (55.2%)	0.530
REM sleep latency, min (*n* = 133)	4.1 ± 3.0	3.9 ± 2.9	4.8 ± 3.0	0.111
**PSG**				
TST, min	459.3 ± 58.8	461.4 ± 58.7	452.1 ± 59.5	0.383
Sleep latency, min	5.5 ± 8.8	6.0 ± 9.6	3.7 ± 4.9	0.033
REM sleep latency, min	61.3 ± 70.0	60.2 ± 65.4	65.1 ± 85.2	0.683
Nocturnal SOREMP	55 (40.7%)	43 (41.0%)	12 (40.0%)	0.537
Sleep efficiency, %	90.3 ± 6.7	90.4 ± 6.8	89.9 ± 6.3	0.715
WASO, min	47.1 ± 43.9	47.8 ± 47.3	44.3 ± 29.3	0.617
N1, %	17.7 ± 12.2	16.8 ± 11.5	20.7 ± 14.1	0.242
N2, %	50.7 ± 11.7	49.9 ± 11.9	53.5 ± 10.6	0.165
N3, %	9.5 ± 9.1	10.8 ± 9.1	5.0 ± 7.4	<0.001
REM, %	21.7 ± 6.9	22.0 ± 6.6	20.9 ± 8.2	0.454
Arousal index, /h	15.7 ± 12.0	14.7 ± 12.2	19.5 ± 10.6	0.016
AHI, /h	6.6 ± 9.7	6.2 ± 10.5	7.8 ± 6.5	0.023
Mean SpO2, %	95.7 ± 1.8	95.8 ± 1.9	95.5 ± 1.6	0.258
Minimum SpO2, %	89.8 ± 4.3	90.2 ± 4.5	88.7 ± 3.6	0.016
PLMI, /h (*n* = 129)	5.2 ± 12.8	4.6 ± 12.3	7.5 ± 14.2	0.973

Data are presented as mean ± standard deviation or number (%). Abbreviations: MSLT, multiple sleep latency test; SOREMP, sleep-onset REM period, PSG, polysomnography; TST, total sleep time; REM, rapid eye movement; WASO, wakefulness after sleep onset; AHI, apnea–hypopnea index; SpO2, peripheral arterial oxygen saturation; PLMI, periodic limb movement index.

**Table 3:** Results of correlation analysis with age at onset of narcolepsy.

Variables	Coefficient *	*p*
**MSLT**		
REM sleep latency, min	**0.241**	**0.005**
Mean sleep latency, min	0.099	0.255
No. of SOREMPs	−0.047	0.590
**PSG**		
TST, min	−0.144	0.095
Sleep latency, min	−0.041	0.640
REM sleep latency, min	−0.058	0.505
Sleep efficiency, %	−0.030	0.729
WASO, min	−0.018	0.837
N1, %	0.090	0.299
N2, %	0.089	0.306
N3, %	**−0.359**	**<0.001**
REM, %	0.059	0.494
Arousal index, /h	**0.200**	**0.020**
AHI, /h	0.127	0.142
PLMI, /h	0.007	0.936
**Clinical**		
BMI, kg/m^2^	0.054	0.534
ESS score	0.043	0.622
PSQI score	−0.050	0.612

* Spearman’s correlation coefficient (ρ). Bold font indicates statistical significance (*p* < 0.05). Abbreviations: MSLT, multiple sleep latency test; REM, rapid eye movement; SOREMP, sleep-onset REM period; BMI, body mass index; ESS, Epworth Sleepiness Scale; PSQI, Pittsburgh Sleep Quality Index; PSG, polysomnography; TST, total sleep time; WASO, wakefulness after sleep onset; AHI, apnea–hypopnea index; PLMI, periodic limb movement index.

**Table 4:** Multiple linear regression analysis of REM sleep latency on the MSLT.

	Univariate			Multivariate		
Variables	β	95% CI	*p*	β	95% CI	*p*
Age at onset, y	0.069	0.011–0.127	0.021	0.049	0.004–0.095	0.033
Female (vs. male)	0.320	−0.716–1.357	0.542	0.249	−0.564–1.063	0.545
BMI, kg/m^2^	−0.174	−0.283–−0.066	0.022	−0.106	−0.194–−0.018	0.018
NT1 (vs. NT2)	−2.742	−3.649–−1.834	<0.001	−1.083	−1.968–−0.199	0.017
**MSLT**						
No. of SOREMPs	−1.475	−1.864–−1.085	<0.001	−0.921	−1.335–−0.507	<0.001
Mean sleep latency, min	0.644	0.427–0.861	<0.001	0.258	0.044–0.472	0.018
**PSG**						
Sleep efficiency, %	0.134	0.061–0.207	<0.001	0.058	−0.003–0.119	0.064
WASO, min	−0.018	−0.029–−0.007	0.002			
N1, %	−0.082	−0.122–−0.043	<0.001			

β denotes the unstandardized coefficient. Abbreviations: CI, confidence interval; BMI, body mass index; MSLT, multiple sleep latency test; SOREMP, sleep-onset REM period; PSG, polysomnography; WASO, wakefulness after sleep onset.

**Table 5:** Cox proportional hazard models for positive SOREMPs in the first nap trial of the MSLT.

	Univariate			Multivariate		
Variables	HR	95% CI	*p*	HR	95% CI	*p*
Age at onset, y	0.966	0.941–0.991	0.009	0.955	0.929–0.982	0.001
Female (vs. male)	0.792	0.534–1.175	0.792	0.868	0.582–1.296	0.490
BMI, kg/m^2^	1.033	0.991–1.077	0.122			
NT1 (vs. NT2)	1.718	1.166–2.532	0.006	1.093	0.718–1.665	0.678
ESS score	0.998	0.950–1.048	0.938			
PSG sleep efficiency, %	0.997	0.969–1.025	0.829			
No. of SOREMPs	2.121	1.719–2.617	<0.001	2.155	1.707–2.720	<0.001
Mean sleep latency, min	0.858	0.771–0.954	0.005	0.983	0.878–1.100	0.764

Abbreviations: SOREMP, sleep-onset REM period; MSLT, multiple sleep latency test; HR, hazard ratio; CI, confidence interval; BMI, body mass index; NT1, narcolepsy type 1; NT2, narcolepsy type 2; ESS, Epworth Sleepiness Scale; PSG, polysomnography.

## Data Availability

Deidentified data are available from the corresponding author (K.I.Y.) upon reasonable request.

## Supplementary Materials

The following supporting information can be downloaded at https://www.mdpi.com/article/10.3390/jcm14124379/s1, Table S1: Multivariate Cox proportional hazards model for positive SOREMPs in the first nap trial of MSLT according to narcolepsy type.

## Abbreviations

The following abbreviations are used in this manuscript:

BMI	body mass index
CSF	cerebrospinal fluid
EDS	excessive daytime sleepiness
ESS	Epworth Sleepiness Scale
MSLT	multiple sleep latency test
NT1	narcolepsy type 1
NT2	narcolepsy type 2
PSG	polysomnography
PSQI	Pittsburgh Sleep Quality Index
REM	rapid eye movement
SOREMP	sleep-onset rapid eye movement period
