# Supplementary material for: Impact of Age at Narcolepsy Onset on Sleep-Onset REM Periods in the Multiple Sleep Latency Test
Source: J Clin Med. 2025 Jun 19;14(12):4379. doi: 10.3390/jcm14124379 (PMC12194353; doi:10.3390/jcm14124379)
Supplement: Supplementary file 1 [file jcm-14-04379-s001.zip › jcm-3660705-supplementary.pdf]

Table S1. Multivariate Cox proportional hazards model for positive SOREMP in the first nap trial of MSLT according to narcolepsy type

	NT1 (n = 70)			NT2 (n = 65)		
**Variables**	**HR**	**95% CI**	**P**	**HR**	**95% CI**	**P**
Age at onset, y	0.957	0.923-0.994	0.021	0.946	0.905-0.988	0.013
Sex, female	0.818	0.479-1.390	0.454	0.833	0.511-1.717	0.833
No. of SOREMPs	2.086	1.480-2.939	< 0.001	2.030	1.461-2.820	< 0.001
Mean sleep latency, min	0.905	0.740-1.108	0.334	1.028	0.901-1.173	0.686

Abbreviations: SOREMP, sleep-onset REM period; MSLT, multiple sleep latency test; HR, hazard ratio; CI, confidence interval; NT1, narcolepsy type 1; NT2, narcolepsy type 2.
